# Thiobarbituric Acid Reactive Substances (TBARS) Levels in Schizophrenia Patients: A Comparative Study of ECT and Pharmacological Treatment

**DOI:** 10.1192/j.eurpsy.2026.10719

**Published:** 2026-07-31

**Authors:** Y. Darben Azarsız, E. S. Ahi Üstün, H. Kaya, A. Civan Kahve, P. Dumancı, A. Uzdoğan, E. Göka

**Affiliations:** 1 Psychiatry, University of Health Sciences, Ankara City Hospital; 2 Psychiatry, Mamak State Hospital; 3 Psychiatry, Gazi University Faculty of Medicine; 4 Biochemistry, University of Health Sciences, Ankara City Hospital, Ankara, Türkiye

## Abstract

**Introduction:**

Electroconvulsive therapy (ECT) is employed as an effective and safe treatment modality for schizophrenia(Pompili M, *et al*. Schizophr Res 2013; 146 1–9). Oxidative stress has been implicated in the pathophysiology of schizophrenia(Bitanihirwe BK, Woo T-UW. Neurosci Biobehav Rev 2011; 35 878–93). Thiobarbituric Acid Reactive Substances (TBARS) are widely used as a marker of lipid peroxidation(Grignon S, Chianetta JM. Prog Neuropsychopharmacol Biol Psychiatry 2007; 31 365–9), yet the impact of ECT on TBARS levels remains unclear.

**Objectives:**

This study aims to compare TBARS levels in inpatients with schizophrenia who received ECT (n=18) versus those treated pharmacologically without ECT (n=20), and to examine their associations with clinical parameters.

**Methods:**

38 inpatients diagnosed with schizophrenia were included. 18 patients received ECT in addition to pharmacotherapy, while 20 received pharmacotherapy only. Blood samples were collected on the first and last days of hospitalization to measure serum TBARS. Sociodemographic and clinical data were recorded at admission and CGI (Clinical Global Impression), PANSS (Positive and Negative Symptom Scale) scales were applied. Analyses were conducted with SPSS v28. Non-normally distributed data (Shapiro–Wilk) were tested using Mann–Whitney U, Wilcoxon signed-rank, chi-square, Spearman correlation, and multiple linear regression; with significance at p<0.05. (The ethics committee approval number is TABED 1-24-306)

**Results:**

Of the 38 patients, 18 received ECT plus medication, while the remaining 20 received medication alone. No significant differences were found between groups in terms of age, total duration of disorder, years of education, PANSS, CGI scores, or suicide history (all p>0.05). However, clozapine use was higher in the ECT group (χ²=4.374, p=0.036). TBARS levels showed no significant change from admission to discharge in either group (ECT group: 756.5 vs. 768.5 nmol/L; Z=-1.136, p=0.256; pharmacotherapy group: 644.0 vs. 693.5 nmol/L; Z=-1.512, p=0.130). There was also a similar percentage change in TBARS levels between groups (%9.6 vs. %15.3; Z=-0.380, p=0.704). Negative correlations were found between TBARS levels and both age (ρ=-0.441, p<0.001) and the duration of the disorder (ρ=-0.333, p<0.05). Regression analysis showed suicide history as the only significant predictor of TBARS levels (β=-0.402, p=0.013).

**Conclusions:**

TBARS levels did not significantly change following either ECT or pharmacological treatment in schizophrenia inpatients, suggesting limited impact of ECT on oxidative stress as measured by lipid peroxidation. However, associations with age, illness duration, and history of suicide suggest potential relevance of oxidative stress markers in subgroups of patients.

**Disclosure of Interest:**

None Declared

